# Urgent X‐Rays in Children With Unexplained Haematemesis Help Rule Out Button Battery Ingestion

**DOI:** 10.1111/apa.70244

**Published:** 2025-07-26

**Authors:** Malni Irene, Marin Maura, Barbi Egidio, Amaddeo Alessandro

**Affiliations:** ^1^ Department of Medicine, Surgery and Health Sciences University of Trieste Trieste Italy; ^2^ Institute for Maternal and Child Health IRCCS ‘Burlo Garofolo’ Trieste Italy

**Keywords:** button battery ingestion, diagnostic delay, hematemesis, oesophageal injury, unwitnessed ingestion

## Abstract

**Aim:**

The ingestion of foreign bodies, particularly button batteries, is a significant concern in paediatric care, especially in children under 4 years of age. This study aims to review unwitnessed button battery ingestion in infants and toddlers, considering the serious complications and the higher risk in children under 2 years old.

**Method:**

A literature review was conducted on studies published between 1983 and 2025 using the terms ‘disk battery’, ‘button battery’, ‘ingestion’, and ‘unwitnessed’. Fifteen studies were included, reporting a total of 41 cases of unwitnessed button battery ingestion.

**Results:**

The median age of patients was 18 months. Common symptoms included dysphagia, vomiting, fever, drooling and hematemesis. The average time to presentation at the emergency department was 72 h. Diagnostic delays were often due to initial misidentification of the battery as a coin. Radiographic imaging of the chest and abdomen identified the battery in 92% of cases, highlighting the diagnostic value of X‐rays.

**Conclusion:**

Prompt chest and abdominal X‐rays are recommended for children under 4 years presenting with unexplained hematemesis to rapidly identify button battery ingestion, minimise diagnostic delays and improve clinical outcomes.

**Summary**

- Unwitnessed button battery ingestion poses a serious risk in children under four, especially those under two.
- A review of 15 studies with 41 cases found common symptoms such as dysphagia and hematemesis, with frequent diagnostic delays caused by misidentification as coins.
- Chest and abdominal X‐rays are essential for rapid diagnosis, reducing delays and improving clinical outcomes in affected children.

## 1 Introduction

Foreign body ingestion in the paediatric population is not uncommon and may involve various small objects, some harmless and others highly dangerous, such as button batteries. An increasing number of household items contain button batteries, and children under 4 years old are particularly at risk of ingesting them without parental awareness.

Risk factors for severe complications include age younger than 2 years, battery exposure duration exceeding 6 h and a ≥ 20 mm battery diameter [1].

Fatal outcomes in these cases are often linked to the formation of an aortoesophageal fistula or fistulas involving other major mediastinal vessels, leading to massive haemorrhage. When ingestions are witnessed, emergency protocols for battery removal are immediately activated. However, even in such cases, clinical complications can be fatal or cause significant disability. The situation becomes more complicated when they are not witnessed, as diagnostic delay significantly impacts patient outcomes [2].

When presented with unexplained dysphagia and/or drooling, a chest X‐ray should always be considered to rule out a radio‐opaque foreign body. Most of the existing paediatric protocols for the evaluation of hematemesis include X‐ray imaging when foreign body ingestion is suspected. However, this is not consistently regarded as a screening tool for all patients with unexplained hematemesis. Furthermore, the indication often applies only to chest X‐ray, with abdominal radiography frequently overlooked. It is also rarely specified that the imaging should also include the cervical portion of the oesophagus. In this context, there is no clear consensus on the optimal approach to X‐ray imaging for haematemesis in children [3, 4, 5].

Identification of a button battery significantly alters management, as these cases require time‐critical care in a hospital capable of providing multispecialty support—including cardiothoracic surgical expertise and potential need for bypass. Additionally, consideration must be given to pre‐removal ingestion of honey or sucralfate, and to intraoperative irrigation with acetic acid if no visible perforation is present [6].

The aim of this study was to conduct a comprehensive review of the literature regarding unwitnessed and non–self‐reported button battery ingestion in infants. For each case included, we documented the presenting symptoms, whether radiographic imaging was performed, and if it contributed to the diagnosis.

## 2 Method

We conducted the research based on the following keywords: (disk battery OR button battery OR battery) AND (ingestion OR swallowing OR foreign body) AND (unwitnessed OR asymptomatic OR occult). We reviewed MEDLINE and Scopus for literature covering studies published between 1983 and February 2025. Only studies published in English were taken into consideration.

**FIGURE 1:**
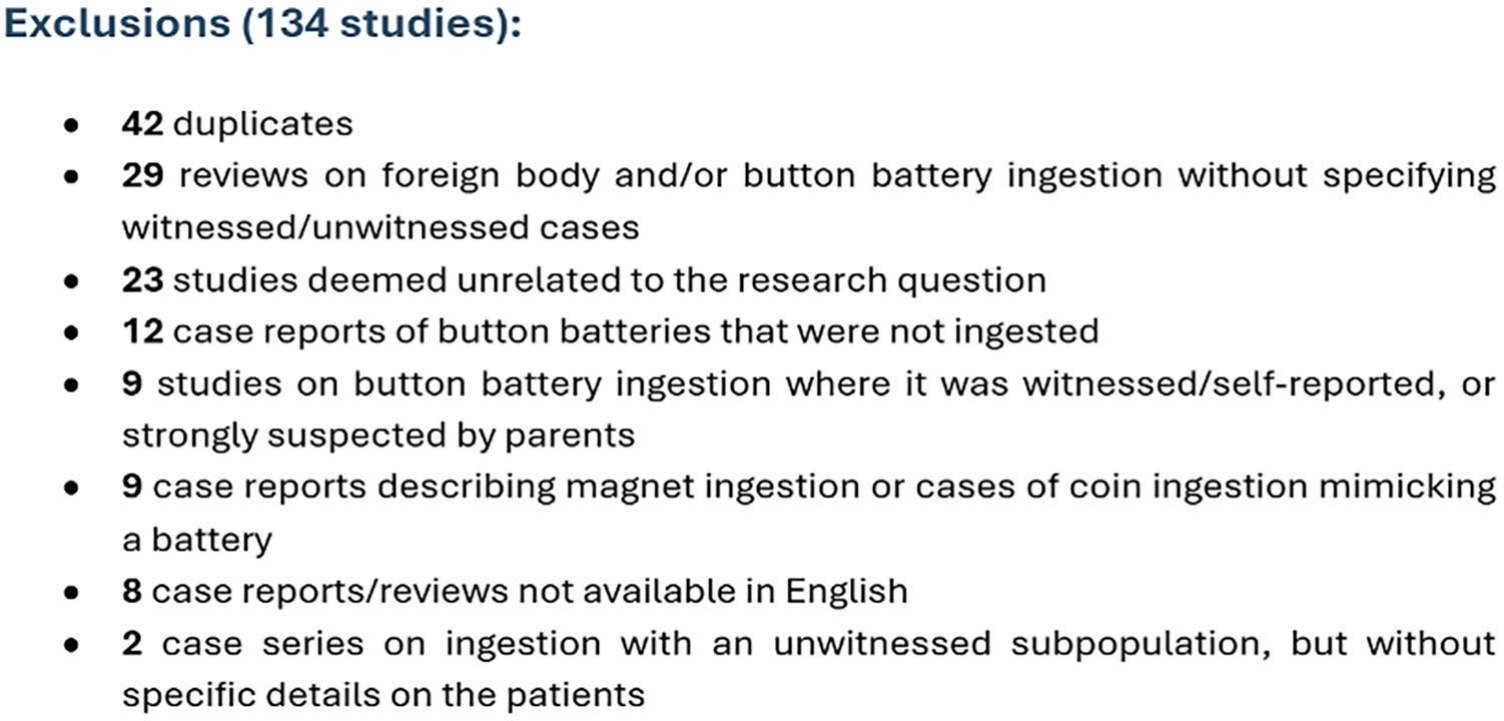
Studies excluded.

We identified 149 studies, but after applying exclusion criteria, we excluded 133 (see Figure 1). Two case series were not incorporated in the sample for this review, as they reported unwitnessed ingestion but did not provide specific patient characteristics. Ultimately, 15 studies were included in the analysis, comprising 7 case series and 8 case reports. From these 15 selected studies, we identified 41 patients after excluding those whose ingestion was witnessed. Among the case series, one patient appeared in two separate studies, but we counted him only once to avoid duplication (Figure S1).

## 3 Results

The median age of the 41 patients (53.6% male) was 18 months, with a minimum age of 4 months and a maximum of 8 years. These findings were consistent with previous literature, which reported similar demographic patterns (Table 1).

**TABLE 1:** Summary of patient demographics and relevant clinical information.

		Sex	Age (months)	Sintoms at presentation	Time from (in hours)	Complication		Localization of BB	RX performed?	Exam of diagnosis
1	Konzett et al. [7]	F	24	Hematemesis, epistaxis	Unknown	Aortoesophageal fistula	Death	Upper oesophagus	Not performed	Authopsy
		M	14	Hematemesis	Unknown	Aortoesophageal fistula	Death	Lower gastrointestinal tract	Yes	RX
2	Oftring et al. [8]	F	19	Stridor	2160	Tracheal extrinsic compression, oesophageal stricture	Survive	Upper oesophagus	Yes	RX
3	Karnecki et al. [9]	F	15	Epistaxis	Unknown	Aortoesophageal fistula	Death	Mid oesophagus	Not performed	Authopsy
4	Revathi et al. [10]	F	36	Hematemesis, abdominal pain	Not reported	Oesophageal ulcer	Survive	Stomach	Yes	RX
5	Littlehales et al. [11]	M	15	Distress, drooling, vomiting	7	Oesophageal ulcer	Survive	Upper oesophagus	Yes	RX
6	Leinwald et al. [12]	F	16	Irritability, hematemesis	2	Oesophageal ulcer, hemorragy	Death	Colon	Yes	RX
		F	24	Thoracic pain, coughing, vomiting, hematemesis	3	Oesophageal ulcer	Death	Distal oesophagus	Yes	RX
		F	18	Hematemesis, abdominal pain	24	Aortoesophageal fistula	Death	Unknown	No	Authopsy
		M	24	Fever, sore throat, vomiting, anorexia	48	Oesophageal ulcer	Survive	Upper oesophagus	Yes	RX
		M	11	Fever, drooling, coughing, anorexia	24	Oesophageal ulcer, vocal cord paralysis, tracheo‐oesophageal fistula	Survive	upper oesophagus	Yes	RX
	
		F	15	Coughing, fever, anorexia	216	Oesophageal ucer, oesophageal stricture, femoral thrombosis (due to cardiac catheterism)	Survive	Mid oesophagus	Yes	RX
F	20	Drooling, fever, vomiting, neck pain, dysphagia	72	Oesophageal ulcer, oesophageal stricture	Survive	Upper oesophagus	Not performed	CT scan
7	Young et al. [13]	F	20	Coughing, fever	168	Oesophageal necrosis, spondylodiscitis	Survive	Upper oesophagus	Yes	RX
8	Simonin et al. [14]	M	16	Coughing, distress, stridor	48	Bilateral vocal cord paralysis	Survive	Upper oesophagus	Yes	RX
9	Litowitz et al. [15]	M	30	Vomiting, fever, dysphagia, inability to talk	24	Tracheo‐oesophageal fistula	Death	Upper oesophagus	Not reported	Not reported
		F	16	Vomiting, fever, tachypnea, dehydratation	96	Aortoesophageal fistula, hydropneumothorax	Death	Upper oesophagus	Not reported	Not reported
		M	25	Cianosis, tachypnea, dysphagia, anorexia	120	Aortoesophageal fistula	Death	Upper oesophagus	Not reported	Not reported
10	Hamilton et al. [16]	M	19	Abdominal pain, coughing, letargy, anorexia	24	Aortoesophageal fistula, bilateral vocal cord paralysis	Death	Distal oesophagus, stomach	Yes	RX
		F	9	Dispnea, stridor, vomiting	Not reported	Bilateral vocal cord paralysis	Survive	Upper oesophagus	Yes, but at second look, initially confused with a coin	RX
11	Abdollah et al. [17]	M	13	Chocking, vomiting, anorexia	1512	Oesophageal ulcer, oesophageal stricture	Survive	Upper oesophagus	Not performed	Flexible endoscopy
		N	17	Chocking, cough, drooling, vomiting	120	Oesophageal ulcer	Survive	oesophagus	No	Operative endoscopy
12	Escobar et al. [18]	M	24	Abdominal pain, stipsis	168	Perforated Meckel's diverticulum	Survive	Lower gastrointestinal tract	Yes, but at second look, initially confused with a coin	RX
13	Srivastava [19]	N	23	Irritability, dysphagia, odinophagia	168	Oesophageal necrosis	Survive	Mid oesophagus	Yes	RX
14	Yardeni et al. [20]	F	9	Distress, dysphagia, melena, fever	168	Tracheo‐oesophageal fistula	Survive	Upper oesophagus	Not reported	Not reported
		M	36	Dyspnea	504	Tracheo‐oesophageal fistula, oesophageal stricture	Survive	Upper oesophagus	Not reported	Not reported
		M	4	Distress, dysphagia, fever	30	Tracheo‐oesophageal fistula	Survive	Upper oesophagus	Not reported	Not reported
		M	5	Distress	288	Tracheo‐oesophageal fistula	Survive	Upper oesophagus	Not reported	Not reported
		M	18	Vomiting, dysphagia, irritability	696	Oesophageal ulcer, oesophageal stricture	Survive	Upper oesophagus	Not reported	Not reported
		M	36	Dysphagia	48	Perforazione esofagea, oesophageal stricture	Survive	Upper oesophagus	Not reported	Not reported
	
		M	24	Cyanosis, tachypnea, dysphagia	120	Aortoesophageal fistula	Survive	Upper oesophagus	Not reported	Not reported
		F	18	Dysphagia	72	Oesophageal perforation, oesophageal stricture	Survive	Upper oesophagus	Not reported	Not reported
F	16	Dysphagia	672	Tracheo‐oesophageal fistula	Survive	Upper oesophagus	Not reported	Not reported
15	Dorterler et al. [21]	M	2	Fever, recurrent pulmonary infection	960	Tracheo‐oesophageal fistula	Survive	Upper oesophagus	Yes	RX
		M	77	Hypersalivation	4	Unknown	Survive	Upper oesophagus	Yes	RX
		M	14	Vomiting, hypersalivation	Unknown	Unknown	Survive	Mid oesophagus	Yes	RX
		M	39	Dysphagia	24	Oesophageal stricture, unilateral vocal cord paralysis	Survive	Mid oesophagus	Yes	RX
		F	99	Hypersalivation	6	Nessuna	Survive	Upper oesophagus	Yes	RX
		M	8	Hypersalivation	8	Unknown	Survive	Upper oesophagus	Yes	RX
		F	55	Vomiting, hypersalivation	24	Oesophageal necrosis, oesophageal stricture	Survive	Mid oesophagus	Yes	RX
		M	15	Dysphagia, vomiting	4	Oesophageal ulcer, oesophageal stricture	Survive	Distal oesophagus	Yes	RX

Regarding symptom presentation, the median time between symptom onset and arrival at the emergency department was 72 h, with a range spanning from 2 h to over 3 months. The most frequently reported symptoms included dysphagia and vomiting, each observed in 31.7% of cases, corresponding to 13 out of 41 patients, followed by fever (24.4%), drooling (22%), haematemesis (14.6%), dyspnea (14.6%), coughing (14.6%) and anorexia (14.6%) (see Table 1). Remarkably, in this sample, two patients (4.8%) presented with epistaxis. In one of these cases, it was the only symptom, resulting from massive haemorrhage from the oesophagus and not initially recognised as haematemesis [9].

Radiographic examinations were documented as performed in 60.9% of the 41 cases and as not performed in 9.7% of cases. In 92% of the 25 cases in which X‐rays were performed (23 out of 25), the battery was successfully identified. The battery was initially mistaken for a coin in two cases, but the correct diagnosis was achieved upon further image review. In two other cases, X‐rays were not diagnostically helpful. In one instance, the battery was misidentified as a coin, and the correct diagnosis was only accomplished during operative endoscopy [17]. In another case, the diagnosis was established post‐mortem, as the autopsy revealed lesions compatible with button battery ingestion, although the battery itself was never recovered [12].

Among cases in which radiographic examinations were conducted, imaging was limited to the chest in the majority of instances (18/25), whereas abdominal or cervical radiographs were additionally obtained in a minority of cases (2/25 and 1/25, respectively). Isolated abdominal imaging was performed in 2 cases, and isolated cervical imaging in one case. In one case, the type of X‐ray was not specified. The choice appears to be largely driven by the presumed localisation of the presenting symptoms.

## 4 Discussion

Haematemesis is rare in children but it is still the most common clinical presentation of upper gastrointestinal bleeding. The most frequent causes in children aged 1–5 years include erosive esophagitis, gastritis, caustic ingestions, peptic ulcer bleeding and varices. Foreign bodies and caustic substances are less common causes, but early diagnosis can prevent mortality and morbidity and should not be overlooked [22]. Diagnosing button battery ingestion is straightforward with a simple bedside X‐ray, as these foreign bodies are radiopaque, and radiography has a high sensitivity for their detection [23]. In terms of a risk–benefit consideration, the recommendation for routine X‐rays should acknowledge the balance between early diagnosis and potential drawbacks.

In this battery ingestion case series, 14.6% of patients (6 cases) presented with hematemesis. In 4 out of 6 patients, corresponding to 66.6% of those with hematemesis, X‐ray examination was decisive for the diagnosis. In two toddlers presenting with haematemesis, radiographic examination was not performed in one case and negative in the other case; the diagnosis of button battery ingestion was established during autopsy []. With the rising incidence of button battery ingestions, a chest and abdomen X‐ray is increasingly likely to be high yield, and when positive, significantly impacts management.

In 63.4% of this population, the button battery was in the upper oesophagus, underscoring the importance of extending the X‐ray to include the cervical portion of the oesophagus.

This review suggests that a chest and abdominal X‐ray, including the cervical oesophagus, should be performed in any child presenting with unexplained haematemesis.

Another key point highlighted by these data is that hematemesis can present as unrecognised epistaxis. In patients with epistaxis without evidence of bleeding from the nasal cavities, a diagnosis of button battery ingestion should be considered.

### 4.1 Limitations

The main limitation of this review was the small number of available studies with individual case details, and a larger sample size was needed to define the sensitivity and specificity of this method in a screening context.

While the available data may not yet definitively support the routine use of an urgent X‐ray including the entire oesophagus and abdomen in children with unexplained hematemesis, drooling, dysphagia and epistaxis, overall, the findings from this case series provide compelling evidence that such an approach is both clinically justified and potentially critical for timely diagnosis and management.

## Conflicts of Interest

The authors declare no conflicts of interest.

## Supporting information


**Figure S1:** apa70244‐sup‐0001‐FigureS1.docx.

## Data Availability

The data that support the findings of this study are available from the corresponding author upon reasonable request.
