# Supplementary material for: Urgent X‐Rays in Children With Unexplained Haematemesis Help Rule Out Button Battery Ingestion
Source: Acta Paediatr. 2025 Jul 26;114(11):2798–805. doi: 10.1111/apa.70244 (PMC12520257; doi:10.1111/apa.70244)
Supplement: Supplementary file 1 — Figure S1: apa70244‐sup‐0001‐FigureS1.docx. [file APA-114-2798-s001.docx]

Figure S1. “Chart of included and excluded studies”.

*Included studies:* 15

- 7 case series
- 78case reports

*Excluded studies:* 134

- 42 duplicates
- 29 reviews on foreign body and/or button battery ingestion without specifying witnessed/unwitnessed cases
- 23 studies deemed unrelated to the research question
- 12 case reports of button batteries that were not ingested
- 9 studies on buttery battery ingestion where it was witnessed/self-reported, or strongly suspected by parent
- 9 case reports describing magnet ingestion or cases of coins ingestion mimicking a battery
- 8 case reports/reviews not available in English
- 2 case series on ingestion with an unwitnessed subpopulation, but without specific details on the patients

*Total number of studies found:* 149

*Bibliographic search*

Databases: Medline and Scopus

Period: from 1983 to 2025

Keywords: "button battery" OR "disk battery" OR "battery", AND ingestion OR swallowing OR "foreign body", AND unnoticed OR asymptomatic OR occult
